# Measures to Eradicate Foot-and-Mouth Disease

**Published:** 1903-01

**Authors:** D. E. Salmon

**Affiliations:** Chief of Bureau


### MEASURES ADOPTED TO CONTROL AND ERADICATE FOOT-AND-MOUTH DISEASE FROM THE NEW ENGLAND STATES BY THE BUREAU OF ANIMAL INDUSTRY AND STATE AUTHORITIES.

*To Inspectors Engaged in Eradication of Foot-and-Mouth Disease:*

The eradication of foot-and-mouth disease is the most urgent and important problem confronting the live-stock industry at this time. The inspectors and others engaged upon this work are helping to make an imperishable part of the veterinary sanitary history of the country. Should the disease become more prevalent or escape to the West, the condition would constitute a grave calamity which might easily reach national proportions. If the disease is confined to its present area and is exterminated there, great credit will be awarded to all who have had a part in this memorable achievement. It is hoped that every individual inspector, agent, or other employé will realize his own great responsibility in this service and will do all that he can to insure the early and complete eradication of this plague.

For the purpose of explaining and systematizing the work, the following instructions are issued:

Co-operation between the States and the Federal Government. The work in hand will be carried out, so far as possible, in co-operation between the authorities of the States and the Bureau of Animal Industry. In some instances agents of the Bureau of Animal Industry will be given commissions from the Cattle Bureau or the Cattle Commission of a State, so that they may exercise authority that is not directly conferred by the Bureau of Animal Industry. The responsibility of the inspectors and agents is in large degree a joint responsibility, as they will be clothed with authority both from the State and the nation. It is therefore important that all work shall be conducted in full harmony with the State and local inspectors.

*General Plan of Operation.* The general plan of operation will be to locate every diseased animal of the infected place as soon as possible, and then to establish such restrictions as will effectually prevent the transfer of infection. All infectious material must be held in seclusion until it has perished or has been destroyed, that is to say, until the subjects have fully recovered or have been killed and the premises disinfected.

Everyone is encouraged to send to Dr. S. E. Bennett, 147 Milk Street, Boston, Mass., any reports or rumors that he may hear in regard to the probable or possible existence of disease in any new locality.

*Suggestions Regarding Inspections.* In inspecting animals with a view of discovering the existence of foot-and-mouth disease, it should be borne in mind that the acute symptoms causing appreciable illness are of short duration. The cow becomes feverish and depressed not more than a day before the vesicles appear in the characteristic locations. After this the temperature falls to near normal. The vesicles last one or two days, and after they have broken, unless the areas involved are exceptionally large or complications arise, healing starts almost at once. If the vesicles are large or numerous upon the teats or about the hoofs, soreness of these regions may remain for two weeks or more. Therefore, if an inspection is made for the purpose of determining whether a herd has passed through the disease, and in the absence of superficial erosions upon the pad, within the lips, upon the gums or tongue, special weight should be placed upon slight lameness, undue moisture of the skin between the toes, and sore teats.

It may be of value in some instances to apply at the creamery or milk shipping station for information as to the quantity of milk produced at the time it is suspected that the herd was affected. A more or less sudden fall in the yield, lasting for one or two weeks, might be a valuable clue. Much may sometimes be gained by conversation with cattle dealers and live-stock owners in regard to outbreaks in other places.

In sheep and swine the symptoms are about as they are in cattle, but the vesicles are more likely to be confined to the feet. All exposed cloven-footed animals are to be regarded as possible carriers of the disease and are to be quarantined and reported.

*How to Avoid Spreading Infection.* It is of the utmost importance that the inspectors shall not themselves carry infection from place to place. This may be avoided by scrupulous attention to the following precautions:

Each inspector shall be provided with a rubber coat coming within nine inches of the ground, a pair of rubber boots, and a bottle of creolin or some mercuric chloride tablets. He should also have a cotton skull-cap that may be carried in the pocket of the rubber coat. Before going into a stable in which there is any reason to suspect that infection may exist, this special attire shall be put on. Upon coming out, the exposed parts of the coat and boots shall be washed off thoroughly with a 3 per cent. solution of creolin or with mercuric chloride solution, 1:1000. All dust, dirt, and manure shall be removed from the coat and boots in this way. The inspector shall then remove his cap and place it in the pocket of his coat, pack his special clothing in a bag provided for this purpose, and then disinfect his hands. The cap shall be disinfected at the close of each day's work. If so much washing makes the hands sore rubber gloves may be worn, if disinfected carefully each time after use.

*Importance of Absolute Quarantine.* Do not fail to impress upon each person in charge of a quarantined herd the absolute necessity of a strict and complete quarantine. Explain what such a quarantine means. Give to each such person a copy of Circular No. 38, Bureau of Animal Industry, on foot-and-mouth disease. Especially impress the importance of excluding all visitors, and of those who have been about infected cattle or premises keeping away from the stock or premises of others. Remember that dogs and cats must be confined and all stray animals excluded from quarantined premises. Keep all cattle dealers away.

*Reports.* Make reports carefully and promptly. If special conditions of importance arise telephone or telegraph to Dr. S. E Bennett, 147 Milk Street, Boston. Do not fail to report anything that may have a bearing on the origin or the additional distribution of this disease. D. E. SALMON,

Chief of Bureau.

